# Supplementary figures and images for: Altered cardiolipin metabolism is associated with cardiac mitochondrial dysfunction in pulmonary vascular remodeled perinatal rat pups
Source: PLoS One. 2022 Feb 10;17(2):e0263520. doi: 10.1371/journal.pone.0263520 (PMC8830687; doi:10.1371/journal.pone.0263520)

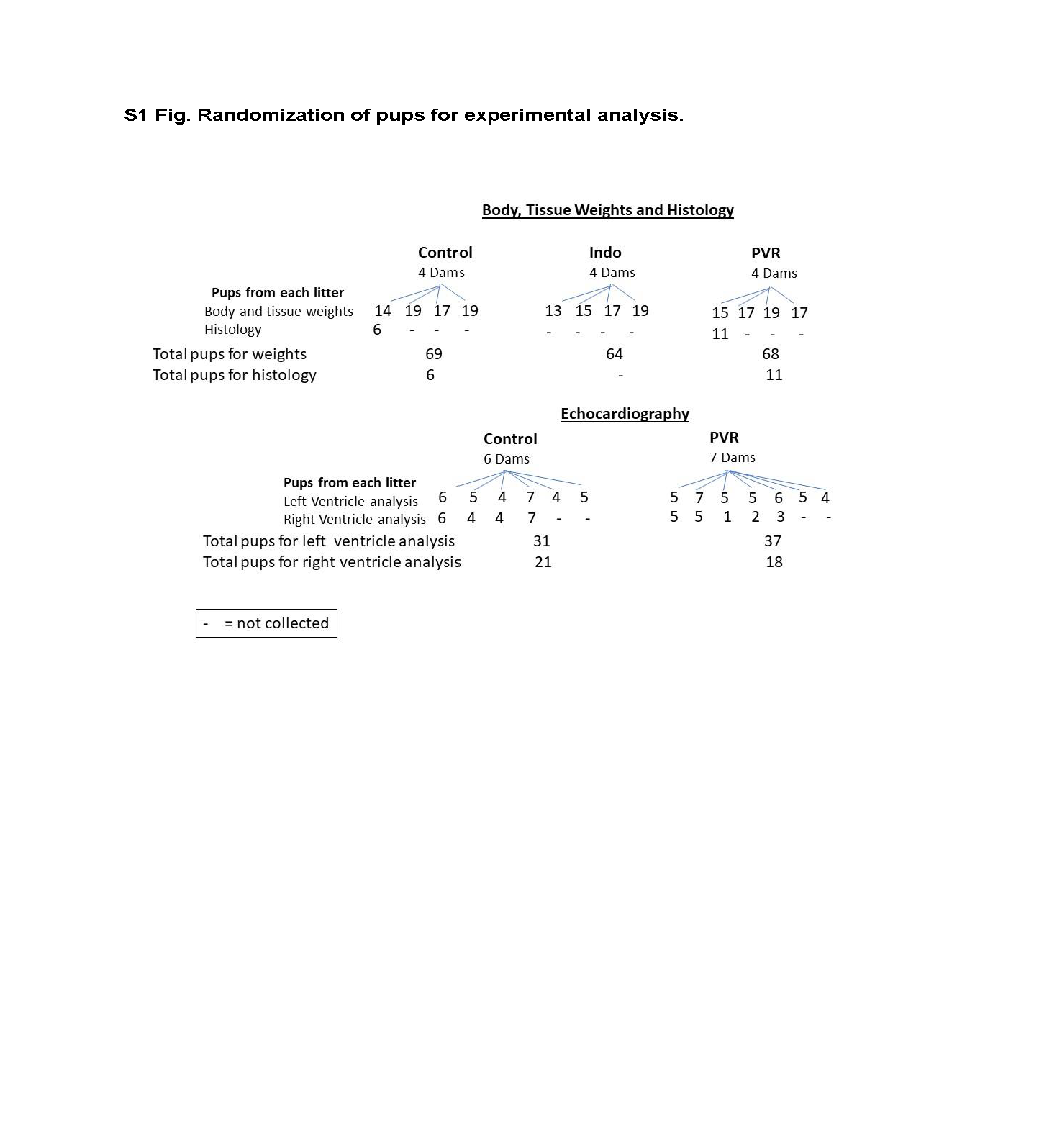

Supplement: S1 Fig — (TIF) [file pone.0263520.s001.tif]

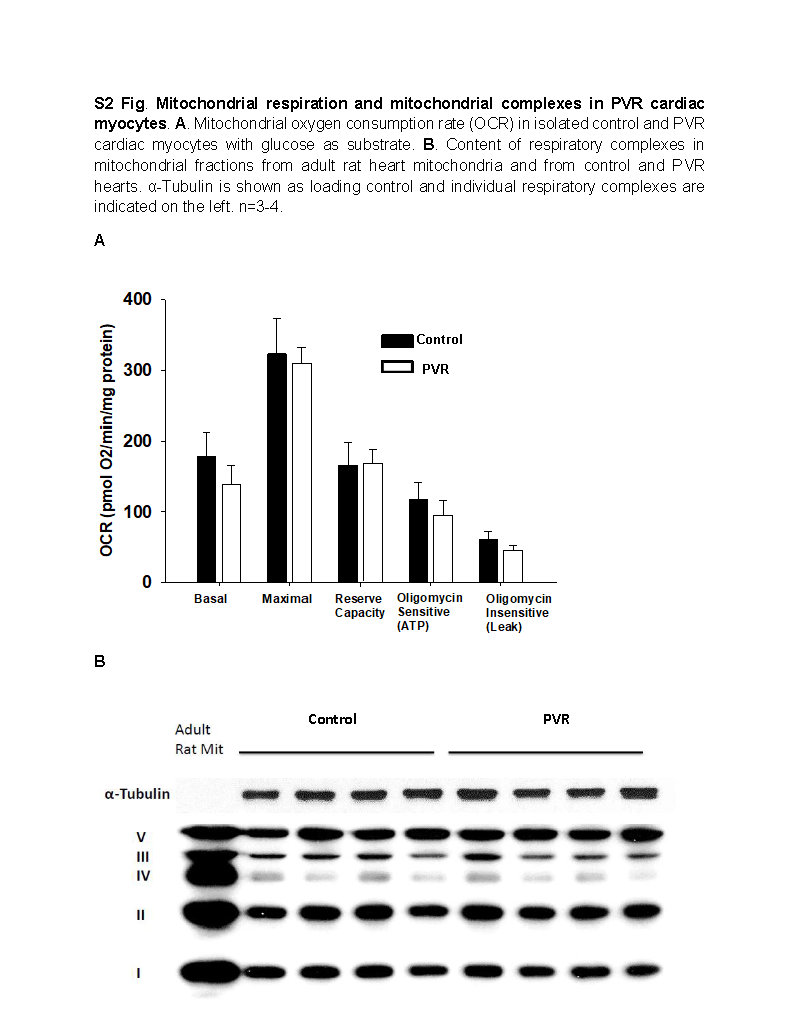

Supplement: S2 Fig — (TIF) [file pone.0263520.s002.tif]

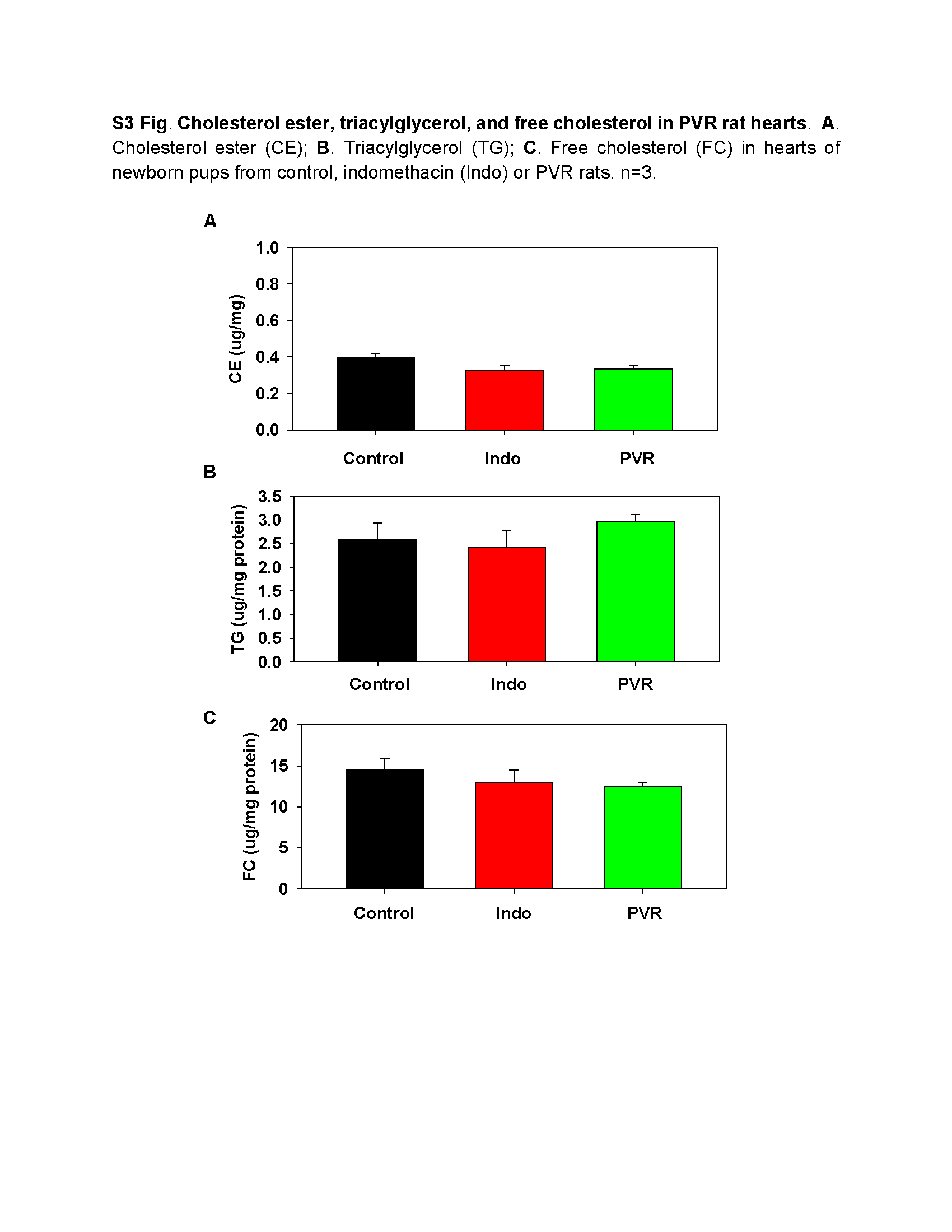

Supplement: S3 Fig — (TIF) [file pone.0263520.s003.tif]
